# Post-transarterial chemoembolization lipiodol migration with obstructive biliopathy

**DOI:** 10.1007/s12664-024-01542-0

**Published:** 2024-02-12

**Authors:** Akhil Mahajan, Rahul Puri, Kiran Mane, Sridhar Sundaram

**Affiliations:** Department of DDCN, Tata Memorial Hospital, Parel, Mumbai 400 012 India

A 67-year-old man with alcoholic cirrhosis had hepatocellular carcinoma in segment 8. He underwent transarterial chemoembolization (TACE) and radiofrequency ablation (RFA). After eight weeks of TACE and RFA, he presented with abdominal pain and progressive distension of abdomen. Contrast-enhanced computed tomography (CECT) showed migration of lipiodol into the terminal common bile duct (CBD) with resultant upstream CBD dilatation with extra-hepatic rupture of right peripheral hepatic duct with an ill-formed biloma (Fig. 1). Endoscopic retrograde cholangiopancreatography (ERCP) was done, which showed leak from right anterior duct on cholangiogram with dilated CBD and filling defects (Fig. 2). After sphincterotomy and balloon sweeps, lipiodol fragment could be retrieved and 10Fr PS was placed following CBD clearance.

**Fig. 1:**
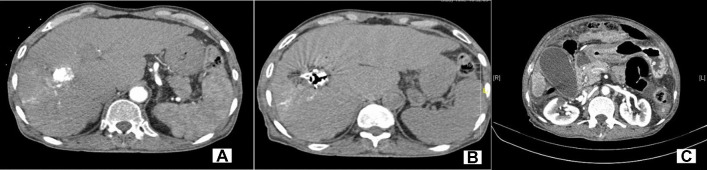
Lipiodol distribution post-transarterial chemoembolization and deposition in the lesion (**A** and **B**) with subsequent migration into terminal common bile duct (CBD) with CBD dilatation and gross distention of the gallbladder (**C**). Diffuse peritoneal fat stranding with ascites and peritoneal enhancement

**Fig. 2:**
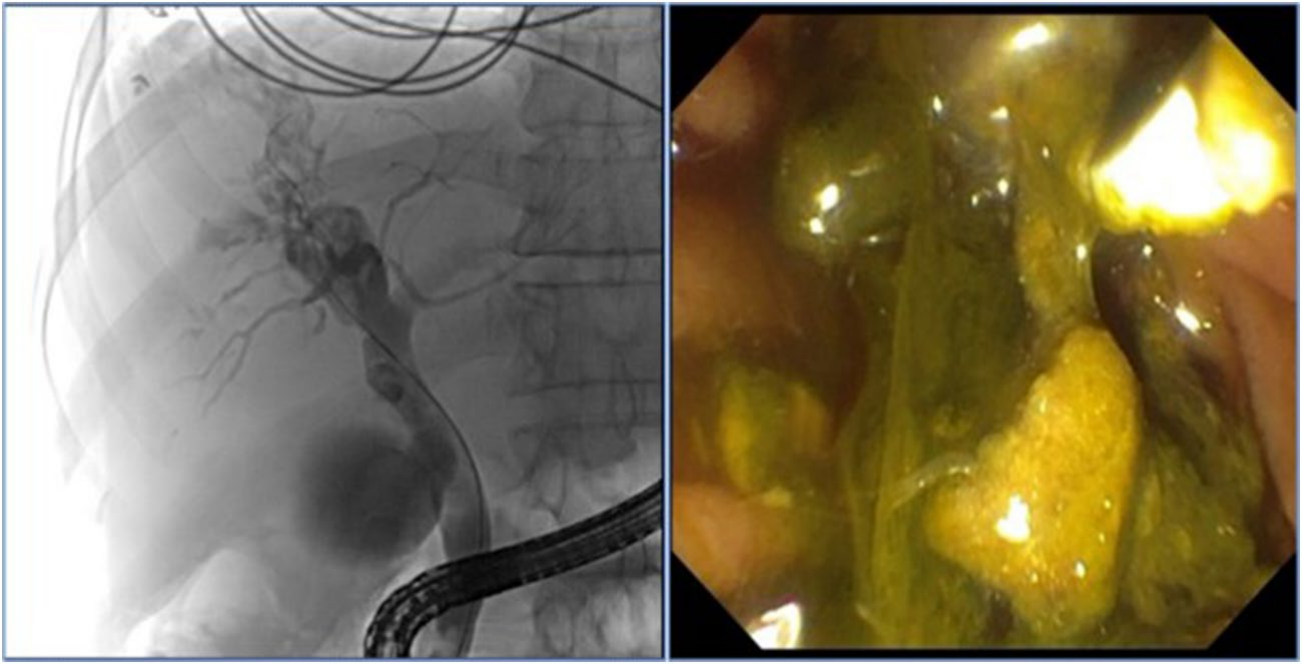
Leak from right anterior duct on cholangiogram and balloon sweeps taken after sphincterotomy, which retrieved lipiodol fragments

Post-TACE lipiodol migration leading to biliary obstruction and bile leak is very rare with very few case reports till date [1]. The goal of endoscopic treatment is to eliminate the trans-papillary pressure gradient, permitting free flow of bile [2]. There is no difference with respect to success rate and adverse events in relation to the diagnosis of bile leak and timing of intervention [3]. We aim to acquaint clinicians with this uncommon complication of a very common procedure in the present era of rising hepatocellular carcinoma prevalence.

## Declarations

### Disclaimer

The authors are solely responsible for the data and the content of the paper. In no way, the Honorary Editor-in-Chief, Editorial Board Members, the Indian Society of Gastroenterology or the printer/publishers are responsible for the results/ findings and content of this article.
